# Supplementary material for: Evaluation of ex vivo drug combination optimization platform in recurrent high grade astrocytic glioma: An interventional, non-randomized, open-label trial protocol
Source: PLoS One. 2024 Jul 26;19(7):e0307818. doi: 10.1371/journal.pone.0307818 (PMC11280195; doi:10.1371/journal.pone.0307818)
Supplement: S2 File — (PDF) [file pone.0307818.s002.pdf]

STUDY PROTOCOL

PROTOCOL TITLE:

Evaluation of ex vivo drug combination optimization platform in recurrent high grade astrocytic glioma

PROTOCOL NUMBER: QP25/05/21

PROTOCOL VERSION: 5

PROTOCOL DATE: 4 Jul 2023

PRINCIPAL INVESTIGATOR:

Dr Andrea Wong[1], Senior Consultant, National University Hospital (NUH)

CO - INVESTIGATORS:

Dr Yvonne Ang[1], Consultant, NUH
A/Prof. Yeo Tseng Tsai[2], Senior Consultant, NUH
Dr Teo Kejia[2], Consultant, NUH
Dr Nga Diong Weng[2], Consultant, NUH
Dr Bolem Nagarjun[2], Senior Resident, NUH
Prof Khong Pek Lan[7], Senior Consultant, NUH
Dr Jocelyn Wong[7], Senior Consultant, NUH
Dr Balamurugan A Vellayappan[9], Senior Consultant, NUH
Prof Goh Boon Cher[1], Senior Consultant, NUH
Prof Lee Soo Chin[1], Senior Consultant, NUH
Dr Chee Cheng Ean[1], Senior Consultant, NUH
A/Prof David Tan[1], Senior Consultant, NUH
Dr Ross Soo[1], Senior Consultant, NUH
Dr Joline Lim[1], Consultant, NUH
Dr Raghav Sundar[1], Consultant, NUH
Dr Matilda Lee[1], Associate Consultant, NUH
Dr Joan Choo[1], Associate Consultant, NUH
Dr Gloria Chan[1], Associate Consultant, NUH
Dr Dinesh Nivedh[2], Senior Resident, NUH

SITE PRINCIPAL INVESTIGATOR

Prof Khong Pek Lan[8], Director, YLLSoM, NUS

SITE PRINCIPAL INVESTIGATOR

Adj A/Prof Low Shiong Wen[10], Senior Consultant, NTFGH

CO - INVESTIGATORS:
Dr Ira Sun[10], Consultant, NTFGH

COLLABORATORS:

A/Prof. Edward Chow Kai Hua[4,5,6], Associate Professor, NUS
Prof. Dean Ho, Director[4,5], NUS
Dr Toh Tan Boon[4,5], Senior Research Fellow, NUS
Dr Ding Lingwen[6], Senior Research Fellow, NUS

[1]: Department of Haematology-Oncology, National University Hospital
[2]: Department of Surgery, Division of Neurosurgery, National University Hospital
[3]: Department of Radiation Oncology, National University Cancer Institute, Singapore
[4]: The N.1 Institute for Health (N.1), National University of Singapore
[5]: The Institute for Digital Medicine (WisDM), National University of Singapore
[6]: Cancer Science Institute of Singapore (CSI), National University of Singapore
[7]: Department of Diagnostic Imaging, National University Hospital
[8]: Clinical Imaging Research Centre (CIRC), National University of Singapore
[9]: Department of Radiation Oncology, National University Hospital
[10]: Department of Neurosurgery, Ng Teng Fong General Hospital

STUDY SITE:

National University Hospital (NUH), National University of Singapore (NUS), Ng Teng Fong General Hospital (NTFGH)

Contents

CONTENTS **3**

1 TITLE: **5**

1.1 OBJECTIVES: 5
1.2 TRIAL DESIGN: 5
1.3 CHOICE OF DOSING REGIMEN 6
1.3.1 Inclusion Criteria *8*
1.3.2 Exclusion Criteria *8*
1.4 STUDY ASSESSMENTS 10
1.5 OUTCOMES MEASURES AND STATISTICAL PLAN 11
1.6 EFFICACY ENDPOINTS 11

2 BACKGROUND **12**

2.1 CLINICAL SIGNIFICANCE AND RATIONALE FOR THE STUDY 12
2.2 PRECLINICAL DATA ON THE APPLICATION OF QPOP TO OTHER MALIGNANCIES. 13
2.3 PRECLINICAL DATA ON THE DEVELOPMENT OF PATIENT DERIVED ORGANOIDS (PDOS) IN HIGH GRADE ASTROCYTIC GLIOMA 14

3 HYPOTHESIS AND OBJECTIVES **14**

3.1 HYPOTHESIS 14
3.2 STUDY ENDPOINTS 16

4 INVESTIGATIONAL PLAN **17**

4.1 SUMMARY OF STUDY DESIGN 17
4.2 CHOICE OF DOSING REGIMEN 18
4.3 SAFETY AND EFFICACY ASSESSMENTS 19
4.4 OVERALL STUDY SCHEMA 19

5 INVESTIGATOR INFORMATION **20**

5.1 STUDY POPULATION AND SUBJECT ENROLMENT 20
5.1.1 Inclusion Criteria *20*
5.1.2 Exclusion Criteria *21*
5.1.3 Withdrawal Criteria *21*
5.1.4 Subject Replacement *22*

6 DOSAGE AND ADMINISTRATION **22**

6.1 STUDY TREATMENT 22

7 STUDY VISITS AND PROCEDURES **22**

8 SPECIAL TESTS **23**

9 SAFETY MEASUREMENTS **24**

9.1 DEFINITIONS 24
9.2 COLLECTING, RECORDING AND REPORTING OF "UNANTICIPATED PROBLEMS INVOLVING RISK TO SUBJECTS OR OTHERS" – UPIRTSO EVENTS TO THE NHG DOMAIN SPECIFIC REVIEW BOARDS (DSRB) 25
9.3 SAFETY MONITORING PLAN 26

10 DATA ANALYSIS **26**

10.1 DATA QUALITY ASSURANCE 26
10.2 DATA ENTRY AND STORAGE 26

11 SAMPLE SIZE AND STATISTICAL METHODS **27**

12 ETHICAL CONSIDERATIONS **27**

12.1 INFORMED CONSENT 27
12.2 IRB REVIEW 27
12.3 CONFIDENTIALITY OF DATA AND PATIENT RECORDS 27

13 RETENTION OF TRIAL DOCUMENTS 28
14 REFERENCES 29
15 APPENDIX 1 STUDY SCHEDULE 31

1 *Title:*

Evaluation of ex vivo drug combination optimization platform in recurrent high grade astrocytic glioma

1.1 Objectives:

Our central hypothesis is that PDOs mimic the biological characteristics of high grade astrocytic gliomas and serve as an ideal platform for the evaluation of drug sensitivities, accurately reflecting the patient's therapeutic response to the drugs. This will enable us to facilitate therapeutic decision-making and personalised anti-cancer therapy. In this study, we will specifically evaluate drug sensitivities/ resistance of high grade astrocytic glioma organoids before and after standard-of-care first line therapy, and match these with treatment outcomes, with the objective of determining if QPOP analysis can effectively predict therapeutic sensitivities as well as determine the optimal treatment at the time of relapse. High grade astrocytic glioma is an ideal tumor type to test our hypothesis because there is currently no standard-of-care second line systemic therapy for this disease. In addition, the blood-brain barrier (BBB) poses significant challenges to the delivery of systemic therapy to brain tumors. This study presents a unique opportunity to examine the relationship between individualized tumoral therapeutic sensitivity, BBB permeability and patient outcomes. We aim to evaluate how BBB permeability evolves over time from first diagnosis to tumor recurrence using Gallium-68 NOTA-Evans Blue (Ga68-NEB) Positron Emission Tomography/ Magnetic Resonance Imaging (PET/MRI) and Dynamic Contrast Enhanced (DCE)-MRI imaging, and will correlate imaging biomarkers with QPOP-derived therapeutic sensitivities, clinical and radiological response as well as patient outcomes.

Specific Aim 1: To establish primary and corresponding temozolomide/radiotherapy (TMZ/RT)-resistant high grade astrocytic glioma patient-derived organoids.

Specific Aim 2: To determine the utility of QPOP (Quadratic Phenotypic Optimization Platform)-derived drug combinations in treating recurrent high grade astrocytic glioma.

Specific Aim 3: To evaluate the use of non-invasive imaging tools (Ga68-NEB PET/MRI and DCE-MRI) to track BBB permeability over time in high grade astrocytic glioma patients, and correlate this with pathological biomarkers, therapeutic response determined by follow up standard MRI and clinical outcomes.

1.2 Trial Design:

This is an interventional, non-randomized, single site study. In the pilot phase of our study, we will aim to enrol 10 patients who will receive QPOP-guided systemic therapy at the time of first high grade astrocytic glioma recurrence. Subjects will be replaced accordingly if their QPOP analysis is unsuccessful or its results do not lead to therapeutic decision making.

Pre-screening phase:

Patients from NUH and NTFGH (involve in only Pre-screening phase) will be approached for pre-screening consent at the time of first suspected diagnosis of a high grade astrocytic glioma and tumor will be harvested at the time of initial surgery/ biopsy for the generation of PDOs. Once the histological diagnosis of high grade astrocytic glioma is confirmed and the patient is planned for adjuvant temozolomide and radiotherapy, our prescreening cohort will undergo baseline standard MRI plus Ga68-NEB PET/MRI and DCE-MRI imaging prior to chemoradiotherapy **(Radiological timepoint 1).** If patients are admitted to the ward, they will not be sent for the Ga68-NEB PET/MRI and DCE-MRI imaging. These scans should be arranged as outpatient scans only after they are discharged from the ward. In addition, tumor may be harvested for any subsequent procedure that patient might require for brain tumor treatment. Study imaging and further PDO

generation will not take place if the patient does not meet the histological criteria or will not be receiving standard adjuvant temozolomide/ radiotherapy. It is estimated that we will need to pre-screen approximately 20 patients in order to achieve our objective of enrolling 10 recurrent high grade glioma patients treated with QPOP-guided systemic therapy.

Patients will then undergo standard-of-care treatment for high grade astrocytic glioma with adjuvant concurrent temozolomide and radiotherapy (TMZ/RT)[1], and will be evaluated by the primary oncologist routinely with clinical examination, laboratory tests and regular neuro-imaging (performed as standard clinical care and not part of the study). High grade astrocytic glioma organoids will be generated from resected tumour samples and QPOP analyses of the treatment-naïve high grade astrocytic glioma organoids will be performed (**QPOP 1).** To model TMZ/RT-resistant cells in the setting of recurrent high grade astrocytic glioma, these treatment-naïve organoids will be subjected to chronic exposure of temozolomide, and ionizing radiation. QPOP analyses will be performed on these resistant high grade astrocytic glioma organoids to identify specific drug combinations to guide clinical management at the time of first relapse (**QPOP 2**).

Main study:

At the time of documented tumor recurrence, eligible patients will be reassessed for suitability to participate in the main study and approached for their informed consent to enter this phase of the study. At this juncture, a small proportion of patients will be deemed suitable for a second operation. If this is the case, high grade astrocytic glioma organoids will also be generated from the recurrent high grade astrocytic glioma tumour and subject to QPOP analyses (**QPOP 3**). During the main study, patients will receive QPOP-guided systemic therapy for the treatment of their relapsed high grade glioma and will be assessed regularly for safety and efficacy of this therapy. In addition, patients will undergo standard MRI plus investigational Ga68-NEB PET/MRI and DCE-MRI imaging prior to and 8 weeks (+/- 1 week) after QPOP-guided systemic therapy (**Radiological timepoints 2 and 3**, respectively). If patients are admitted to the ward, they will not be sent for the Ga68-NEB PET/MRI and DCE-MRI imaging. These scans should be arranged as outpatient scans only after they are discharged from the ward. Subsequent to this, radiological assessment of their disease will revert to standard clinical protocols with routine standard MRI imaging.

1.3 Choice of dosing regimen

We will have two *in vitro* drug screening panels. The “clinical care panel” (**Panel 1**) comprises anti-cancer agents which have previously been evaluated in recurrent high grade astrocytic glioma: SN-38, lomustine, temozolomide, vincristine, procarbazine, etoposide, carboplatin, cisplatin, cyclophosphamide, docetaxel, capecitabine, vinorelbine.[1-13] All of these drugs have been determined to have sufficient penetration across the blood-brain barrier and have known adverse event profiles. QPOP will be used to derive top-ranking drug combinations, which will then be validated *in vitro*. The “experimental panel” (**Panel 2**) comprises anti-cancer agents which have promising activity in high grade astrocytic glioma but are not yet routinely used in high grade astrocytic glioma patients: Geftinib, osimertinib, vemurafenib, everolimus, sunitnib, regorafenib, selinexor, marizomib, abemaciclib, ivosidenib, olaparib, metformin. The results of this panel will not be used in the care of our enrolled patients; instead they are hypothesis-generating and intended for us in designing future trials.

The drug combinations generated by QPOP can be divided into 3 categories: 1) combination regimens with published data in the setting of gliomas **(category 1)**; 2) combination regimens

where there is published data on intracranial activity and anti-glioma effect of the individual agents (either as monotherapy or in combination with other agents), and where there is published safety data on the combination in non-glioma settings **(category 2)**; and 3) novel drug combinations that have not been assessed for clinical efficacy **(category 3)**.

We will apply the following **guidelines for the selection of drug regimen** in individual patients:

1. If QPOP analysis results of QPOP 1, 2 and 3 are all available at the time the patient is due to start systemic therapy for the treatment of recurrent high grade astrocytic glioma (i.e., 4-6 weeks after surgery), they will be used in the following order of preference: QPOP 3> QPOP 2> QPOP 1, provided other eligibility criteria for the use of that particular combination's results are met.
2. We will make every effort to use category 1 combinations, but will consider category 2 combinations should the QPOP analysis not generate any category 1 combinations. Decisions to choose category 2 combinations vs defaulting to monotherapy (see point 3) will be discussed with the study team. Doses of drug combinations used should be as close as possible to those used in the published literature. We will not use novel drug combinations (category 3) that have not previously been assessed for clinical safety.
3. If the QPOP combination therapy results do not fall within the criteria for usage, monotherapy may be considered. Temozolomide monotherapy will not be encouraged unless strongly supported by QPOP 2 or 3 results, in which case, a dose schedule different from first-line therapy should be used.
4. Bevacizumab is an FDA approved drug for the treatment of recurrent high grade astrocytic glioma (Avastin (bevacizumab) injection, Genetech, Inc, December 2017), and may be used in combination with lomustine[11], temozolomide[10,11], irinotecan[10], carboplatin[7] and etoposide[7] for the treatment of high grade astrocytic glioma. However, bevacizumab is not included in the drug screening panel because our current PDO models lacks vasculature, hence the functional testing of anti-angiogenic drugs is irrelevant. In order to be relevant to real-world practice, the study will allow the addition of bevacizumab to any of the above agents, provided that investigators adhere as closely as possible to the regimens in the referenced studies. The addition of bevacizumab will not be permitted if the QPOP results suggest an effective combination where there is no published data for the addition of bevacizumab to that particular treatment combination. Deviations from existing data may be permitted only with the agreement of the study team.

The study team will meet to review the QPOP analysis results to agree on the validity of the data and determine the recommended treatment at the time of relapse for each patient on the study. As an additional safety measure, the study team will also conduct a combined review for each patient at the time of their first post-treatment radiologic assessment, to ensure the appropriateness of therapy based on the efficacy and toxicity of the treatment selected.

Subject Population:

Male and female subjects aged 21 years and above with suspected high grade astrocytic glioma planned for surgery/ biopsy followed by adjuvant chemoradiotherapy will be invited to participate in the pre-screening study. Subjects will only be enrolled in the main study if they had pathologically confirmed high grade astrocytic glioma, and received adjuvant treatment comprising standard-of-care therapy with surgery/biopsy followed by temozolomide and radiotherapy. In addition, they are required to have had sufficient tumor tissue available for PDO generation at baseline. We aim to enrol 10 patients who will receive QPOP-guided chemotherapy at the time of first high grade astrocytic glioma recurrence. In order to achieve this, we expect to pre-screen

approximately 20 subjects. Subjects will be replaced accordingly if their QPOP analysis is unsuccessful or its results do not lead to therapeutic decision making. These subjects will not be included in our study endpoint analysis. This study is expected to take 18-24 months to complete.

Written informed consent for entry into the study will be obtained prior to any study specific procedure. All eligibility criteria and consent forms will be checked before treatment is initiated.

1.3.1 Inclusion Criteria

Subjects may be included in the study only if they meet **all** of the following criteria:

Pre-screening:

1) Patients 21 years of age or older, with ECOG performance status 0 to 2, and with a life expectancy of more than 3 months with suspected high grade astrocytic glioma, fit for treatment comprising standard-of-care therapy with adjuvant temozolomide and radiotherapy if the diagnosis of high grade astrocytoma is pathologically confirmed.
2) Signed informed consent obtained before any study specific procedure. Subjects must be able to understand and be willing to sign the written informed consent.
   * Patients will be enrolled at the time of initial surgery but study imaging and further PDO generation will not take place if the patient is subsequently found not to meet the histological criteria or will not be receiving standard adjuvant temozolomide/ radiotherapy.

All subsequent criteria apply to the main study only:

1) Patients 21 years of age or older, with ECOG performance status 0 to 2, and life expectancy of more than 3 months with pathologically confirmed high grade astrocytic glioma, having undergone first-line standard-of-care therapy with surgery/biopsy followed by temozolomide and radiotherapy. Subjects with truncated adjuvant chemoradiotherapy may be enrolled at the Principal Investigator's discretion.
2) Documented tumor progression based on standard clinical, radiological or histological criteria, and deemed suitable for second line systemic therapy.
3) Sufficient tumor tissue available for PDO generation at baseline and at least one available or pending QPOP result.
4) Adequate organ function as defined by:
   1. Bone marrow function
      i. Haemoglobin ≥ 9g/dl
      ii. Absolute neutrophil count (ANC) ≥ 1.5 x 10^9/L
      iii. Platelet count ≥ 100 x 10^9/L.
   2. Liver function
      i. Bilirubin ≤ 2.5x upper limit of normal (ULN)
      ii. Alanine transaminase (ALT) and aspartate transaminase (AST) ≤ 2.5x ULN or ≤ 5x ULN if liver metastases are present
      iii. Prothrombin time (PT) within the normal range for the institution.
   3. Renal function
      i. Plasma creatinine <1.5x institutional ULN
5) Capable of swallowing tablets.
6) Recovery from any previous drug- or procedure-related toxicity to National Cancer Institute Common Terminology Criteria for Adverse Events (NCI-CTCAE) version 5.0 Grade 0 or 1 (except alopecia), or to baseline preceding the prior treatment.

1.3.2 Exclusion Criteria (both pre-screening/ main study)

1) Chemotherapy, radiotherapy, surgery, immunotherapy or other therapy within 2 weeks of study entry.

2) Pregnancy or breastfeeding at the point where systemic anti-cancer therapy is initiated. Women of childbearing potential must have a negative pregnancy test at the point where systemic anti-cancer therapy is initiated. Women of childbearing potential and men, must agree to use adequate contraception (barrier method of birth control) while on anti-cancer treatment and until at least 3 months after the last study drug administration.
3) Concurrent cancer which is distinct in primary site or histology from the cancer being evaluated in this study EXCEPT cervical carcinoma in situ, treated basal cell carcinoma, superficial bladder tumours (Ta, Tis & T1) or any cancer curatively treated less than 5 years prior to study entry.
4) Patients with leptomeningeal dissemination of disease and/or pure spinal high grade gliomas will be excluded.
5) Kidney disease which would clinically disqualify the subject from serial MRI scans with gadolinium contrast.
6) Previous history of allergy to MRI contrast agents.

1.4 Study Assessments

Clinical (within 2 weeks of study enrolment and at the start of each cycle of treatment):

- Medical history and physical examination, evaluation of performance status (ECOG)
- Adverse event assessment (CTCAE v 5.0)

Radiologic (pre-screening phase: prior to chemoradiotherapy; main study: prior to and 8 weeks (+/- 1 week) after QPOP-guided systemic therapy):

- Standard MRI brain +/- perfusion as indicated
- Ga68-NEB PET/MRI
- DCE-MRI

Laboratory (within 2 weeks of study enrolment and at the start of each cycle of treatment, +/- 3 days window period):

- Laboratory parameters including haematology (FBC), chemistries, others as clinically indicated

Final Study Visit:

Clinical assessment within 30 days from the time of study discontinuation/completion of study treatment.

Special Tests

a) Radiological

Gallium-68 NOTA-Evans Blue (Ga68-NEB) Positron Emission Tomography/ Magnetic Resonance Imaging (PET/MRI) and Dynamic Contrast Enhanced (DCE)-MRI imaging

Standard MRI brain with or without perfusion sequences are typically performed at baseline and after therapy in order to evaluate radiological response. In this study, we plan to add Ga68-NEB (Gallium-68 NOTA-Evans Blue) PET imaging and DCE-MRI imaging to examine BBB permeability and assess in-vivo drug effects on tumor vasculature over time. These studies are planned at 3 timepoints: 1) During the pre-screening phase, following confirmation of histological diagnosis and prior to chemoradiotherapy and 2) during the main study, prior to and 8 weeks (+/- 1 week) after QPOP-guided systemic therapy. Radiological biomarkers will be correlated with radiological response based on follow up with routine MRI and clinical outcomes as well as therapeutic sensitivities.

Radiomics

MRIs obtained at initial diagnosis and subsequent scans will be subjected to radiomic feature extraction, and correlated with spatially annotated histopathological biopsies opportunistically obtained as part of standard-of-care surgical resection both at initial diagnosis (chemotherapy- and radiotherapy-naïve) and at recurrence. Annotation of biopsy site would be done on T1-stereotaxy and pathology images would be correlated with spatial radiomic features and clustering. In addition, part of the biopsy will be used to generate high grade astrocytic glioma organoids, and tested for chemosensitivity towards both temozolomide and the optimal drug combination the participant will be treated with. These spatial properties of the high grade astrocytic glioma biopsies will be used for model building for prediction of post-chemotherapy recurrence.

b) Histopathology

Paraffin-embedded blocks of high grade astrocytic glioma tumour will be stained to assess DNA damage response (ɣH2AX, RAD51), growth factor receptors (EGFR, PDGFR) and hypoxia markers (HIF1A) in relation to blood vessels and axons. Molecular analyses of TP53, ATRX, IDH1, IDH2, BRAF, and EGFRvIII mutations, MGMT methylation status, and 1p19q co-deletion would be performed as part of routine care.

c) Plasma biomarkers

Approximately Fifteen to Eighteen millilitres of blood will be collected together with routine blood tests for plasma biomarkers including circulating tumor DNA, proteomics and DNA methylation at these time points. These will be stored in the NUH tissue repository for future analysis.

- Prescreening - At first MRI scan before chemoradiotherapy
- Main study – At baseline and 8-week post QPOP guided chemotherapy

1.5 Outcomes Measures and Statistical Plan

This is an interventional, non-randomized, single site study. The primary purpose of this study is not hypothesis testing, but to assess the feasibility of QPOP-guided therapy for recurrent high grade astrocytic glioma to be used in a larger scale study. Therefore, this study does not have a formal sample size, but rather, a set benchmarks to determine feasibility. We plan to enrol a total of 10 patients who receive QPOP-guided chemotherapy at the time of first high grade astrocytic glioma recurrence. We expect this to be sufficient for determining the feasibility of a phase II trial in which there will be formal sample size calculations for the achievement of PFS6 $\geq$ 30%.[35] In addition, a phase II study will only be considered feasible if there is successful organoid generation and QPOP analysis in > 50% of samples collected. In this study, subjects will be replaced accordingly if their QPOP analysis is unsuccessful or its results do not lead to therapeutic decision making.

1.6 Efficacy Endpoints

Primary clinical endpoint:

i. **Six-month progression-free survival (PFS 6).** PFS is defined as the time from the start of study treatment to documented progression of disease or death; PFS6 refers to the percentage of patients who are alive and free of high grade astrocytic glioma progression at 6 months.

Secondary clinical endpoints:

ii. **Radiological response assessments at follow up MRI.** The determination of radiographic response is as per the Response Assessment in Neuro-Oncology (RANO) criteria. [14]

iii. **Twelve-month overall survival (OS12).** OS is defined as the length of time from the start of study treatment, that patients diagnosed with the disease are still alive. OS12 refers to the percentage of patients who are alive at 12 months.

iv. **Haematological and non-haematological toxicities.** As defined by National Cancer Institute Common Terminology Criteria for Adverse Events (NCI CTCAE) v5.0

2 BACKGROUND

High grade astrocytic glioma is one of the most common and aggressive adult brain malignancies with limited therapeutic options. Despite a combination of advanced maximal surgical resection, radiation, and chemotherapy treatment, the mean survival of high grade astrocytic glioma patients remains at about 12 months. In addition, almost all high grade astrocytic glioma patients inevitably develop tumor recurrence and there is no standard treatment for recurrent high grade astrocytic glioma patients, making high grade astrocytic glioma one of the hardest challenges in cancer precision therapy.

Therefore, developing and evaluating therapeutic platforms that can provide clinicians with additional therapeutic sensitivity information at the patient-specific level may improve therapeutic outcomes in high grade astrocytic glioma patients, particularly in the recurrence setting. Quadratic Phenotypic Optimization Platform (QPOP) determines the ranking of drug combination response in a biological model system such as patient-derived organoids, and has been successfully applied in haematological malignancies such as multiple myeloma and lymphomas. Due to the challenges in reliably generating clinically meaningful patient-derived organoids in solid tumors, QPOP has not been applied in solid tumors. Recently, we have successfully cultured several high grade astrocytic glioma organoids in vitro. This proposal aims to test the feasibility of QPOP application in high grade astrocytic glioma, by first establishing clinically relevant high grade astrocytic glioma organoid models, followed by the utility of QPOP-derived drug combinations in high grade astrocytic glioma. We hypothesize that optimal QPOP-derived drug combinations from clinically relevant high grade astrocytic glioma organoids improve outcomes of recurrent high grade astrocytic glioma patients. Specifically, we will generate unique matched pair of high grade astrocytic glioma organoids with drug- and radio-resistance to model the inevitable tumor recurrence in high grade astrocytic glioma to provide clinically meaningful patient-derived models. Moving forward, we will determine the drug sensitivity of QPOP-derived drug combinations for high grade astrocytic glioma patients in drug- and radio-resistant high grade astrocytic glioma organoids as well as high grade astrocytic glioma organoids at recurrence if the same patient undergoes repeat surgery. Our study will identify and validate QPOP-guided drug combinations as a second line treatment for recurrent high grade astrocytic glioma patients. Our work will justify the application of QPOP for personalized cancer therapy in high grade astrocytic glioma patients.

2.1 Clinical Significance and Rationale for the Study

High grade astrocytic glioma are among the most devastating human adult solid malignancies. Despite advanced surgical intervention with adjuvant radiation and chemotherapy, high grade astrocytic glioma patients typically have a mean survival period of 15 months following diagnosis.[15] In addition, high grade astrocytic glioma tumors demonstrate vast intra- and inter-tumor heterogeneity that confer treatment resistance which eventually result in tumor recurrence in more than 90% of all high grade astrocytic glioma patients.[16,17] Currently, there is no standard treatment for recurrent high grade astrocytic glioma and the option of repeat surgical intervention is limited to about 25% of all recurrent patients.[18] Clearly, there is an urgent need to develop better therapeutic strategies to improve patient outcome. One of the major obstacles for therapeutic development is the lack of clinically relevant models. At present, U87MG, U251, and T98G are some of the most commonly used two-dimensional (2D) cell research models due to their ease of access and growth. However, 2D cell cultures present limitations due to the lack of tumor heterogeneity and cell-cell/matrix interactions that are present in primary patient tumor tissues.[19] Despite the establishment of the National Cancer Institute 60 Panel (NCI-60) cell lines in an attempt to capture the heterogeneous nature of nine different cancer types, these 2D cell cultures are still unable to comprehensively represent the diversity which cancer patients exhibit, and is currently being phased

out.[20] Studies comparing the molecular profiles of cell lines with primary tumors have reported that conventional cell lines are unable to represent all cancer subtypes.[21] Recently, patient-derived organoids (PDOs) have been shown to faithfully preserve and maintain heterogeneity of several primary cancer types including high grade astrocytic glioma and are being used to evaluate drug sensitivity and their associated genomic profiles.[22,23] PDOs have been shown to retain the molecular profiles of the parental tumors and can be effectively used to investigate drug responses and mechanisms ex vivo.[24] Thus, human PDOs represent more clinically relevant models compared to conventional cell line-based models, with the ability to recapitulate disease heterogeneity for more accurate studies of drug responses. Identifying the most suitable patient-specific drug combinations remains a challenge due to the complex molecular networks that contribute to feedback mechanisms of drug resistance and compensatory oncogenic drivers that limit efficacy of targeted inhibitors. Current biomarker-based approaches in predicting drug sensitivity and clinical outcome are appropriate for select subpopulations and specific drugs but are not broadly applicable.[25] These approaches are largely limited to monotherapy and cannot identify patient-specific drug combinations, nor do they consider other genomic alterations that are unaccounted for by the specific biomarkers used.

2.2 Preclinical Data on the Application of QPOP to Other Malignancies.

To address this critical deficit in combination therapy, we have developed an experimental-analytical hybrid platform, Quadratic Phenotypic Optimization Platform (QPOP) that can rank potential drug combination response in biological model systems. We initially applied QPOP towards identifying novel drug combinations against bortezomib- resistant multiple myeloma.[26] As we have improved the efficiency of the QPOP platform and applied QPOP towards primary patient samples and patient-derived organoids, it has become clear that QPOP may be useful as a clinical decision support platform that may be able to suggest clinically actionable and suitable patient-specific drug combinations derived from drug sensitivity tests using patient-derived materials. Our previous work in hematological malignancies suggest that drug combination rankings and drug sensitivity predictions by QPOP from ex vivo drug combination tests on primary patient samples can accurately identify patient-specific therapeutic options (**Figure 1**). For instance, QPOP was applied towards a hepatosplenic gamma-delta T-cell lymphoma (HSTCL) patient who experienced continual disease progression despite 6 lines of treatment. Following QPOP analysis of primary patient tumor cells, QPOP identified an effective combination of bortezomib/panobinostat that had previously been clinically trialed in T-cell lymphoma patients.[27] Treatment using QPOP-derived therapy resulted in complete and durable response.[28] Patient-specificity of this approach is highlighted in that QPOP did not recommend the same combination for every T-cell lymphoma patient sample, but rather identified a variety of combinations and therapeutic options from within a 12-drug set.

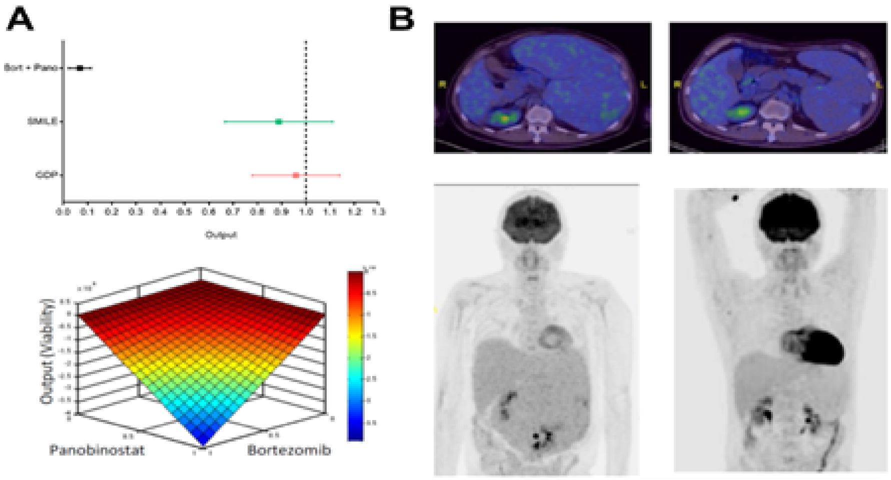

Figure 1: *Ex vivo* drug combination QPOP prediction in T-cell lymphomas. (**A**) Drug combination rankings and predicted outputs in a TCL patient identified Bortezomib/ Panobinostat as the highest ranked therapeutic option. (**B**) Treatment with QPOP-predicted combination Bortezomib/ Panobinostat resulted in complete response.

2.3 Preclinical Data on the Development of Patient Derived Organoids (PDOs) in high grade astrocytic glioma

Three-dimensional (3D) organoid culture systems have been shown to capture the molecular and phenotypic features of the original parental tumors in various organs, including high grade astrocytic glioma. We have successfully cultured several high grade astrocytic glioma high grade astrocytic glioma high grade astrocytic glioma organoids in vitro and measured their growth and stemness characteristics. Importantly, we showed that high grade astrocytic glioma organoids contain a mix of both stem and non-stem cells (**Figure 2**), demonstrating the ability to retain the stemness and cellular heterogeneity of parental tumors. This is important as the recapitulation of complex cancer phenotypes provide more accurate predictions of the therapeutic potential of new treatments.

Using this methodology, we will extend the QPOP application to high grade astrocytic glioma patients and expect to yield important information regarding feasibility of QPOP as a clinical decision support platform as well as the diversity, frequency, and identity of patient-specific drug combinations across a range of brain tumor patients.

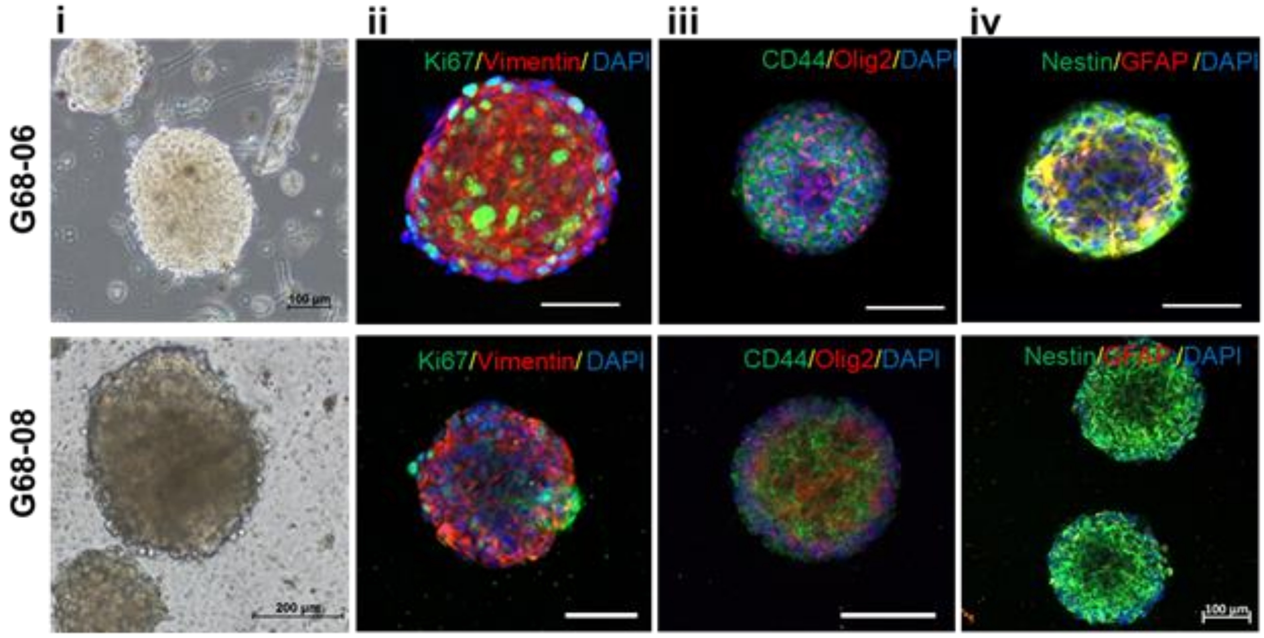

Figure 2: GBM organoids demonstrate proliferative and stem-like characteristics *in vitro*. (i) Brightfield and (**ii-iv**) immunofluorescence images of two GBM organoid cultures (G68-06 and G68-08) demonstrating the presence of proliferative (Ki-67), invasiveness (Vimentin), and cancer stemness (CD44, Olig2, Nestin) markers. Glial fibrillary acidic protein (GFAP) indicates the glial origin of the tumor. Scale bar = 100 µm, unless otherwise stated.

3 HYPOTHESIS AND OBJECTIVES

3.1 Hypothesis

Our central hypothesis is that PDOs mimic the biological characteristics of high grade astrocytic glioma s and serve as an ideal platform for the evaluation of drug sensitivities, accurately reflecting the patient's therapeutic response to the drugs. This will enable us to facilitate therapeutic decision-making and personalised anti-cancer therapy. In this study, we will specifically evaluate drug sensitivities/ resistance of high grade astrocytic glioma organoids before and after standard-of-care first line therapy, with the objective of determining if QPOP analysis can effectively predict therapeutic sensitivities at the time of relapse. high grade astrocytic glioma is an ideal tumor type to test our hypothesis because there is currently no standard-of-care second line systemic therapy for this disease.

In addition, the blood-brain barrier (BBB) poses significant challenges to the delivery of systemic therapy to brain tumors. This study presents a unique opportunity to examine the relationship between individualized tumoral therapeutic sensitivity, BBB permeability and patient outcomes. We aim to evaluate how BBB permeability evolves over time from first diagnosis to tumor recurrence using Gallium-68 NOTA-Evans Blue (Ga68-NEB) Positron Emission Tomography/ Magnetic Resonance Imaging (PET/MRI) and Dynamic Contrast Enhanced (DCE)-MRI imaging, and will correlate imaging biomarkers with QPOP-derived therapeutic sensitivities, clinical and radiological response as well as patient outcomes.

Specific Aim 1: To establish primary and corresponding temozolomide/radiotherapy (TMZ/RT)-resistant high grade astrocytic glioma patient-derived organoids. High grade astrocytic glioma organoids can be readily established from primary patient high grade astrocytic glioma tissues either by encapsulating minced high grade astrocytic glioma tissues in matrigel or as free-floating clusters high grade astrocytic glioma tissues in specific organoid medium. Using the established methods, we will generate unique matched pairs of high grade astrocytic glioma organoids with TMZ/RT resistance to model the inevitable tumor recurrence in high grade astrocytic glioma to provide clinically meaningful patient-derived models for subsequent analysis.

Specific Aim 2: To determine the utility of QPOP (Quadratic Phenotypic Optimization Platform)-derived drug combinations in treating recurrent high grade astrocytic glioma. While promising results from ex vivo drug sensitivity using QPOP with patient-derived cells from multiple myeloma and lymphoma has been demonstrated, it has not yet been successfully applied in solid tumors largely due to the challenges in establishing clinically meaningful patient-derived organoids. Here, using the well-established high grade astrocytic glioma organoid culture systems and the derivation of TMZ/RT-resistant high grade astrocytic glioma organoids, we will determine the drug sensitivity of QPOP-derived drug combinations for high grade astrocytic glioma patients, both in the naïve and TMZ/RT-resistant high grade astrocytic glioma organoids from the same patient.

Specific Aim 3: To evaluate the use of non-invasive imaging tools (Ga68-NEB PET/MRI and DCE-MRI) to track blood brain barrier (BBB**) permeability over time in high grade astrocytic glioma patients, and correlate this with pathological biomarkers, therapeutic response determined by follow up standard MRI and clinical outcomes.** The BBB poses significant challenges to the delivery of therapy to brain tumors. This study presents a unique opportunity to examine the relationship between individualized tumoral therapeutic sensitivity, BBB permeability and patient outcomes. We aim to evaluate how BBB permeability evolves over time from first diagnosis to tumor recurrence [assessed Ga68-NEB (Gallium-68 NOTA-Evans Blue) PET/MRI (Positron Emission Tomography/ Magnetic Resonance Imaging), DCE (Dynamic Contrast Enhanced)-MRI imaging] and will correlate imaging biomarkers with QPOP-derived therapeutic sensitivities, clinical and radiological response as well as patient outcomes. Imaging biomarkers evaluated include measurement of standardised uptake values (SUV) of Ga68-NEB uptake, and perfusion parameters (K_{trans}, V_e, V_p) by DEC-MRI in the tumour.

PET imaging with ^{68}Ga-labeled NOTA (1,4,7-triazacyclononane-N,N′,N″-triacetic acid) conjugated truncated Evans blue (NEB), which complex with serum albumin quickly, and thus most of the radioactivity remain in the blood circulation, allowing for rapid visualization of tracking blood brain barrier (BBB). It has been used clinically to determine blood volume, as a sensitive marker of protein leakage from the vascular lumen in a variety of tissues during inflammation and traumatic injury to evaluate vascular permeability[29], and as a blood-pool imaging agent [30, 31, 32]

We hypothesize and investigate the utility/performance of ^{68}Ga-NEB PET/MRI to track BBB permeability as an alternative to DCE-MRI. The challenges of DCE-MRI as a technique for quantitative imaging include technical challenges with obtaining arterial input function (AIF)

required to derive the quantitative parameters, sensitivity to motion, and poor repeatability for comparison between scans.

^{68}Ga-NEB PET will specifically identify the boundary and determined the molecular volume of BBB permeability in high grade astrocytic glioma patients. Quantitative analysis with the standardized uptake values (SUV) will be obtained that associated with breakdown of the BBB. These PET quantitative values (SUV) and DCE-MRI derived quantitative values (K_{trans}, V_e, V_p) will be evaluated and compared between the imaging modalities and also with response and clinical outcomes. The unique dual-modality imaging PET/MRI offers additional advantages in the evaluation of brain disease. In addition, ^{68}Ga-NEB will offer unique perspectives for targeted radionuclide therapy after labeling the NEB with therapeutic radioisotopes as a theranostic approach.

The dosage of ^{68}Ga-NEB will be 185 ± 37 MBq (5 ± 1 mCi). With an injected dose of 185 MBq (5 mCi), the patient would be exposed to an effective radiation dose of 2.94 mSv, which is much lower than the dose limit of 20 mSv for the second risk category defined by the 2007 International Commission on Radiological Protection [33].

The outcome measures to demonstrate the utility and performance of ^{68}Ga-NEB PET/MRI for its intended purpose will be 1) the visual analysis of PET/MRI characteristics and pattern; 2) molecular lesion volume measured over the VOIs placed over the "hot" lesions of the involved tissues; 3) the standardized uptake values (SUV) of the lesions using the volume of interest (VOI) method and a unified standard. These parameters will be compared to the standard routine radiological follow up MRI assessments per RANO criteria.

We hypothesize that optimal drug combinations for matched naïve and TMZ/RT-resistant high grade astrocytic glioma organoids will be different and that using drug-screening platform results derived from TMZ/RT-resistant high grade astrocytic glioma organoids to select second-line therapy will be more clinically meaningful and improve outcomes of recurrent high grade astrocytic glioma patients. In the event that the patient undergoes surgery at the time of relapse (approximately 30% of our high grade astrocytic glioma population), organoids will be generated from the second surgical specimen, and the drug sensitivity of the QPOP-derived drug combinations compared with those of treatment naïve and TMZ/RT-resistant organoids from the same patient.

3.2 Study Endpoints

The **primary endpoint** of the study is six-month progression-free survival (PFS 6).
PFS is defined as the time from the start of study treatment to documented progression of disease or death; PFS6 refers to the percentage of patients who are alive and free of high grade astrocytic glioma progression at 6 months. This is a common endpoint for clinical trials of second line systemic therapy in high grade astrocytic glioma patients. The PFS6 for recurrent high grade astrocytic glioma ranges from 18-27%.[9]

Secondary clinical endpoints:

i. **Radiological response assessments at follow up MRI.** The determination of radiographic response is as per the Response Assessment in Neuro-Oncology (RANO) criteria. [14]
ii. **Twelve-month overall survival (OS12).** OS is defined as the length of time from the start of study treatment, that patients diagnosed with the disease are still alive. OS12 refers to the percentage of patients who are alive at 12 months.
iii. **Haematological and non-haematological toxicities.** As defined by National Cancer Institute Common Terminology Criteria for Adverse Events (NCI CTCAE) v5.0

4 INVESTIGATIONAL PLAN

4.1 Summary of Study Design

This is an interventional, non-randomized, single site study. In the pilot phase of our study, we will aim to enrol 10 patients who will receive QPOP-guided chemotherapy at the time of first high grade astrocytic glioma recurrence. Subjects will be replaced accordingly if their QPOP analysis is unsuccessful or its results do not lead to therapeutic decision making.

Pre-screening phase:

Patients from NUH and NTFGH (involve in only Pre-screening phase) will be approached for pre-screening consent at the time of first suspected diagnosis of a high grade astrocytic glioma and tumor will be harvested at the time of initial surgery/ biopsy for the generation of PDOs. Once the histological diagnosis of high grade astrocytic glioma is confirmed and the patient is planned for adjuvant temozolomide and radiotherapy, our prescreening cohort will undergo baseline standard MRI plus Ga68-NEB PET/MRI and DCE-MRI imaging prior to chemoradiotherapy **(Radiological timepoint 1).** If patients are admitted to the ward, they will not be sent for the Ga68-NEB PET/MRI and DCE-MRI imaging. These scans should be arranged as outpatient scans only after they are discharged from the ward. In addition, tumor may be harvested for any subsequent procedure that patient might require for brain tumor treatment. Study imaging and further PDO generation will not take place if the patient does not meet the histological criteria or will not be receiving standard adjuvant temozolomide/ radiotherapy. It is estimated that we will need to pre-screen approximately 20 patients in order to achieve our objective of enrolling 10 recurrent high grade glioma patients treated with QPOP-guided systemic therapy.

Patients will then undergo standard-of-care treatment for high grade astrocytic glioma with adjuvant concurrent temozolomide and radiotherapy (TMZ/RT)[1], and will be evaluated by the primary oncologist routinely with clinical examination, laboratory tests and regular neuro-imaging (performed as standard clinical care and not part of the study). High grade astrocytic glioma organoids will be generated from resected tumour samples and QPOP analyses of the treatment-naïve high grade astrocytic glioma organoids will be performed (**QPOP 1).** To model TMZ/RT-resistant cells in the setting of recurrent high grade astrocytic glioma, these treatment-naïve organoids will be subjected to chronic exposure of temozolomide, and ionizing radiation. QPOP analyses will be performed on these resistant high grade astrocytic glioma organoids to identify specific drug combinations to guide clinical management at the time of first relapse (**QPOP 2**).

Main study:

At the time of documented tumor recurrence, eligible patients will be reassessed for suitability to participate in the main study and approached for their informed consent to enter this phase of the study. At this juncture, a small proportion of patients will be deemed suitable for a second operation. If this is the case, high grade astrocytic glioma organoids will also be generated from the recurrent high grade astrocytic glioma tumour and subject to QPOP analyses (**QPOP 3**). During the main study, patients will receive QPOP-guided systemic therapy for the treatment of their relapsed high grade glioma and will be assessed regularly for safety and efficacy of this therapy. In addition, patients will undergo standard MRI plus investigational Ga68-NEB PET/MRI and DCE-MRI imaging prior to and 8 weeks (+/- 1 week) after QPOP-guided systemic therapy (**Radiological timepoints 2 and 3**, respectively). If patients are admitted to the ward, they will not be sent for the Ga68-NEB PET/MRI and DCE-MRI imaging. These scans should be arranged as outpatient scans only after they are discharged from the ward. Subsequent to this, radiological assessment of their disease will revert to standard clinical protocols with routine standard MRI imaging.

4.2 Choice of dosing regimen

We will have two *in vitro* drug screening panels. The "clinical care panel" (**Panel 1**) comprises anti-cancer agents which have previously been evaluated in recurrent high grade astrocytic glioma: SN-38, lomustine, temozolomide, vincristine, procarbazine, etoposide, carboplatin, cisplatin, cyclophosphamide, docetaxel, capecitabine, vinorelbine.[1-13] All of these drugs have been determined to have sufficient penetration across the blood-brain barrier and have known adverse event profiles. QPOP will be used to derive top-ranking drug combinations, which will then be validated *in vitro*. The "experimental panel" (**Panel 2**) comprises anti-cancer agents which have promising activity in high grade astrocytic glioma but are not yet routinely used in high grade astrocytic glioma patients: Geftinib, osimertinib, vemurafenib, everolimus, sunitnib, regorafenib, selinexor, marizomib, abemaciclib, ivosidenib, olaparib, metformin. The results of this panel will not be used in the care of our enrolled patients; instead they are hypothesis-generating and intended for us in designing future trials.

The drug combinations generated by QPOP can be divided into 3 categories: 1) combination regimens with published data in the setting of gliomas **(category 1)**; 2) combination regimens where there is published data on intracranial activity and anti-glioma effect of the individual agents either as monotherapy or in combination with other agents, and where there is published safety data on the combination **(category 2)**; and 3) novel drug combinations that have not been assessed for clinical efficacy **(category 3)**.

We will apply the following **guidelines for the selection of drug regimen** in individual patients:

1. If QPOP analysis results of QPOP 1, 2 and 3 are all available at the time the patient is due to start systemic therapy for the treatment of recurrent high grade astrocytic glioma (i.e., 4-6 weeks after surgery), they will be used in the following order of preference: QPOP 3> QPOP 2> QPOP 1, provided other eligibility criteria for the use of that particular combination's results are met.
2. We will make every effort to use category 1 combinations, but will consider category 2 combinations should the QPOP analysis not generate any category 1 combinations. Decisions to choose category 2 combinations vs defaulting to monotherapy (see point 3) will be discussed with the study team.Doses of drug combinations used should be as close as possible to those used in the published literature. We will not use novel drug combinations (category 3) that have not previously been assessed for clinical safety.
3. If the QPOP combination therapy results do not fall within the criteria for usage, monotherapy may be considered. Temozolomide monotherapy will not be encouraged unless strongly supported by QPOP 2 or 3 results, in which case, a dose schedule different from first-line therapy should be used.
4. Bevacizumab is an FDA approved drug for the treatment of recurrent high grade astrocytic glioma(Avastin (bevacizumab) injection, Genetech, Inc, December 2017), and may be used in combination with lomustine[11], temozolomide[10], irinotecan[10], carboplatin[7] and etoposide[5,7] for the treatment of high grade astrocytic glioma. However, bevacizumab is not included in the drug screening panel because our current PDO models lacks vasculature, hence the functional testing of anti-angiogenic drugs is irrelevant. In order to be relevant to real-world practice, the study will allow the addition of bevacizumab to any of the above agents, provided that investigators adhere as closely as possible to the regimens in the referenced studies. The addition of bevacizumab will not be permitted if the QPOP results suggest an effective combination where there is no published data for the addition of bevacizumab to that

particular treatment combination. Deviations from existing data may be permitted only with the agreement of the study team.

The study team will meet to review the QPOP analysis results to agree on the validity of the data and determine the recommended treatment at the time of relapse for each patient on the study. As an additional safety measure, the study team will also conduct a combined review for each patient at the time of their first post-treatment radiologic assessment, to ensure the appropriateness of therapy based on the efficacy and toxicity of the treatment selected.

4.3 Safety and efficacy assessments

Each subject will be monitored serially by his/ her neuro-oncologist for the duration of QPOP-guided systemic therapy. This includes a clinical examination and a full blood count, liver function and renal panel prior to each cycle of treatment. NCI CTCAE Version 5 will be used for toxicity and dose modifications. The frequency of visits and dose modifications will take place according to institutional guidelines. Investigational neuro-imaging will be performed by magnetic resonance (MR) imaging, (Ga68-NEB PET/MRI and DCE-MRI), at pre-screening and during the main study, prior to and at 8 weeks (+/- 1 week) after QPOP-guided systemic therapy (3 timepoints in total**)**. Imaging on all other occasions will be routine and decided upon by the treating physician. Subjects whose QPOP analysis is unsuccessful or its results do not lead to therapeutic decision making will not be included in the safety and efficacy analysis.

4.4 Overall Study Schema

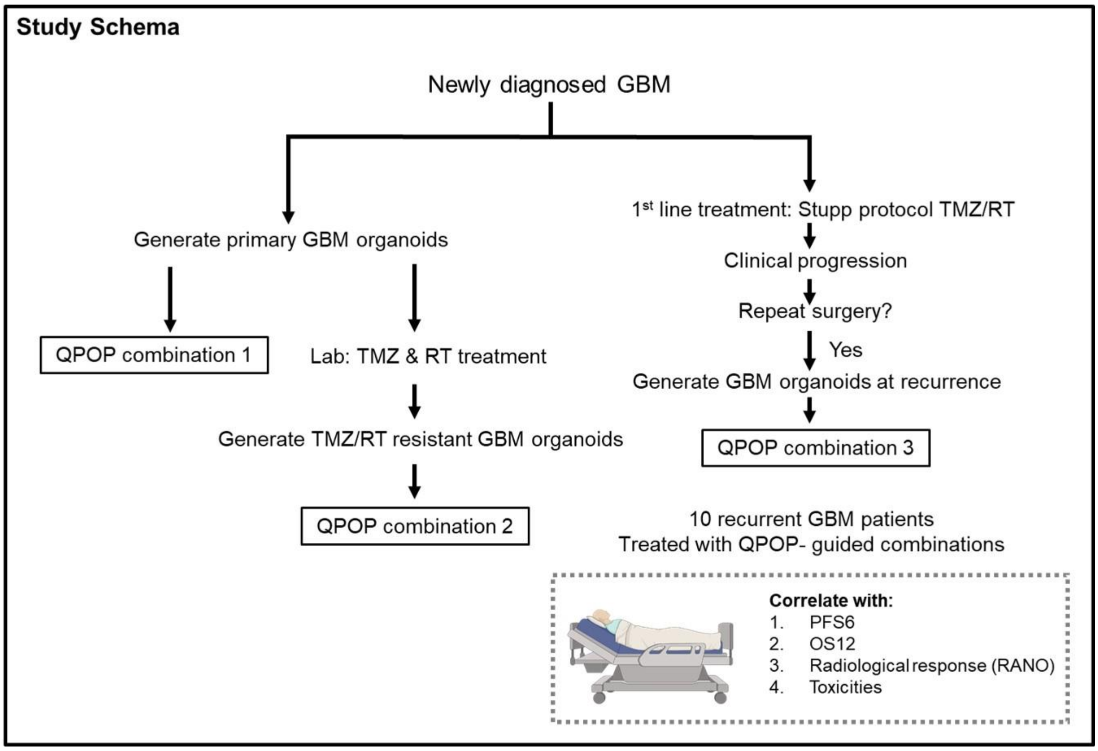

General study schema for QPOP-guided drug combination therapy. Patients will be enrolled to the pre-screening phase of our study at the point of first diagnosis of high grade astrocytic glioma. Established high grade astrocytic glioma PDOs from newly diagnosed patients will be subjected to continuous low dose TMZ and RT as described by the Stupp protocol to model the treatment

received in the clinics. QPOP results will be generated from primary high grade astrocytic glioma PDOs (**QPOP 1**), matched TMZ/RT-resistant high grade astrocytic glioma PDOs (**QPOP 2**). At the point of diagnosis of high grade astrocytic glioma recurrence, patient will be approached for consent for the main protocol if the PDO generation from their primary tumors was successful. If the patient undergoes surgery at the point of recurrence, tissue will be collected for the generation of PDOs at recurrence (**QPOP 3**). QPOP-guided second line systemic treatment will be used in order of preference with available QPOP data from QPOP 3> QPOP 2> QPOP 1. PDOs, patient-derived organoids; TMZ, temozolomide; RT, radiation therapy; QPOP, quadratic phenotypic optimization platform. Figure created with BioRender.com.

5 INVESTIGATOR INFORMATION

The names, titles, and institutions of the investigators are listed in the Contacts for Protocol. If investigators are added after the ethical review board and/or the local regulatory agency have approved the study, these additions will not be considered changes to the protocol, but the Contacts for Protocol will be updated to provide this information.

5.1 Study Population and Subject Enrolment

Male and female subjects aged 21 years and above with suspected high grade astrocytic glioma planned for surgery/ biopsy followed by adjuvant chemoradiotherapy will be invited to participate in the pre-screening study. Subjects will only be enrolled in the main study if they had pathologically confirmed high grade astrocytic glioma, and received adjuvant treatment comprising standard-of-care therapy with surgery/biopsy followed by temozolomide and radiotherapy. In addition, they are required to have had sufficient tumor tissue available for PDO generation at baseline. We aim to to enrol 10 patients who will receive QPOP-guided chemotherapy at the time of first high grade astrocytic glioma recurrence. In order to achieve this, we expect to pre-screen approximately 20 subjects. Subjects will be replaced accordingly if their QPOP analysis is unsuccessful or its results do not lead to therapeutic decision making. These subjects will not be included in our study endpoint analysis. This study is expected to take 18-24 months to complete.

Interim analysis of the PFS6 will be performed after 5 patients have received QPOP-guided chemotherapy. If PFS6 <15% (i.e., none of the patients have achieved 6 months of progression-free survival), this indicates that the trial will likely be futile.[34] The study team will then meet to review potential reasons for this and decide if the trial should be discontinued.

Written informed consent for entry into the study will be obtained prior to any study specific procedure. All eligibility criteria and consent forms will be checked before treatment is initiated.

5.1.1 Inclusion Criteria

Subjects may be included in the study only if they meet **all** of the following criteria:

Pre-screening:

1) Patients 21 years of age or older, with ECOG performance status 0 to 2, and with a life expectancy of more than 3 months with suspected high grade astrocytic glioma, fit for treatment comprising standard-of-care therapy with adjuvant temozolomide and radiotherapy if the diagnosis of high grade astrocytoma is pathologically confirmed.
2) Signed informed consent obtained before any study specific procedure. Subjects must be able to understand and be willing to sign the written informed consent.
   * Patients will be enrolled at the time of initial surgery but study imaging and further PDO generation will not take place if the patient is subsequently found not to meet the histological criteria or will not be receiving standard adjuvant temozolomide/ radiotherapy.

All subsequent criteria apply to the main study only:

1) Patients 21 years of age or older, with ECOG performance status 0 to 2, and life expectancy of more than 3 months with pathologically confirmed high grade astrocytic glioma, having undergone first-line standard-of-care therapy with surgery/biopsy followed by temozolomide and radiotherapy. Subjects with truncated adjuvant chemoradiotherapy may be enrolled at the Principal Investigator's discretion.
2) Documented tumor progression based on standard clinical, radiological or histological criteria, and deemed suitable for second line systemic therapy.
3) Sufficient tumor tissue available for PDO generation at baseline and at least one available or pending QPOP result.
4) Adequate organ function as defined by:
    1. Bone marrow function
        i. Haemoglobin ≥ 9g/dl
        ii. Absolute neutrophil count (ANC) ≥ 1.5 x 10^9/L
        iii. Platelet count ≥ 100 x 10^9/L.
    2. Liver function
        i. Bilirubin ≤ 2.5x upper limit of normal (ULN)
        ii. Alanine transaminase (ALT) and aspartate transaminase (AST) ≤ 2.5x ULN or ≤ 5x ULN if liver metastases are present
        iii. Prothrombin time (PT) within the normal range for the institution.
    3. Renal function
        i. Plasma creatinine <1.5x institutional ULN
5) Capable of swallowing tablets.
6) Recovery from any previous drug- or procedure-related toxicity to National Cancer Institute Common Terminology Criteria for Adverse Events (NCI-CTCAE) version 5.0 Grade 0 or 1 (except alopecia), or to baseline preceding the prior treatment.

5.1.2 Exclusion Criteria (both pre-screening/ main study)

1) Chemotherapy, radiotherapy, surgery, immunotherapy or other therapy within 2 weeks of study entry.
2) Pregnancy or breastfeeding at the point where systemic anti-cancer therapy is initiated. Women of childbearing potential must have a negative pregnancy test at the point where systemic anti-cancer therapy is initiated. Women of childbearing potential and men, must agree to use adequate contraception (barrier method of birth control) while on anti-cancer treatment and until at least 3 months after the last study drug administration.
3) Concurrent cancer which is distinct in primary site or histology from the cancer being evaluated in this study EXCEPT cervical carcinoma in situ, treated basal cell carcinoma, superficial bladder tumours (Ta, Tis & T1) or any cancer curatively treated less than 5 years prior to study entry.
4) Patients with leptomeningeal dissemination of disease and/or pure spinal high grade gliomas will be excluded.
5) Kidney disease which would clinically disqualify the subject from serial MRI scans with gadolinium contrast.
6) Previous history of allergy to MRI contrast agents.

5.1.3 Withdrawal Criteria

- Disease progression,
- Intercurrent illness that prevents further administration of treatment,
- Unacceptable adverse event(s),

- Patient becomes pregnant.
- Patient decides to withdraw from the study, or
- General or specific changes in the patient's condition render the patient unacceptable for further treatment in the judgment of the investigator.

5.1.4 Subject Replacement

Subjects will be replaced accordingly if:

i. Their QPOP analysis is unsuccessful or its results do not lead to therapeutic decision making
ii. The subject who withdraws or discontinues from the study prior to the completion of cycle 1 due to reasons other than toxicity or progressive disease.

6 DOSAGE AND ADMINISTRATION

6.1 Study Treatment

This study does not involve the evaluation of an Investigational Medicinal Product (IMP). All the drugs used in the QPOP drug screening panel have previously been evaluated in high grade astrocytic glioma or other malignancies, with established therapeutic doses. Drug combinations evaluated will be selected only if there is available literature supporting the safety of the combination. Dosages of combination therapy will be in line with published data, unless recommended otherwise by the Study Team. Storage, accountability and prescription of the anti-cancer drugs and their supportive medications will be as per routine clinical use, and in line with institutional guidelines. Toxicities will be monitored at every cycle and dose modifications may be made at the Investigator's discretion, according to institutional guidelines. There are no specific prohibited medications in this study; investigators should exercise due caution when prescribing concomitant medications while patients are on chemotherapy, in line with standard clinical practice.

7 STUDY VISITS AND PROCEDURES

Study Assessments

Clinical (within 2 weeks of study enrolment and at the start of each cycle of treatment):

- Medical history and physical examination, evaluation of performance status (ECOG)
- Adverse event assessment (CTCAE v 5.0)

Radiologic (pre-screening phase: prior to chemoradiotherapy; main study: prior to and 8 weeks (+/-1 week)after QPOP-guided systemic therapy):

- Standard MRI brain +/- perfusion
- Ga68-NEB PET/MRI
- DCE-MRI

Imaging on all other occasions will be routine and decided upon by the treating physician

Laboratory (within 2 weeks of study enrolment and at the start of each cycle of treatment, +/- 3 days window period):

- Laboratory parameters including haematology (FBC), chemistries, others as clinically indicated.

Final Study Visit:
Clinical assessment within 30 days from the time of study discontinuation/completion of study treatment.

Post-Study follow-up:
To obtain meaningful data on survival outcomes, assessments of disease and survival status will be made at regular intervals after patients discontinue from study therapy. Assessments will continue until documented disease progression or death whichever occurs first.

During the post-study follow-up period, information will be collected regarding date of disease progression or death. The study will be closed when, in the opinion of the principal investigator, sufficient data have been obtained for completion of the final study manuscript.

Safety
After a patient discontinues study therapy, the investigator should make every effort to continue to evaluate the patient for delayed toxicity by clinical and laboratory evaluations as clinically indicated. The patient must be followed approximately every 30 days until toxicity resolves.

8 SPECIAL TESTS

All subjects must have at least one tumour lesion that is suitable for biopsy or surgical resection at baseline.

The protocol includes ancillary studies on machine-learning guided radiomics and tumour histopathology, and will be carried out for patients who have given specific consent for these studies.

a) Radiological

Gallium-68 NOTA-Evans Blue (Ga68-NEB) Positron Emission Tomography/ Magnetic Resonance Imaging (PET/MRI) and Dynamic Contrast Enhanced (DCE)-MRI imaging

Standard MRI brain with or without perfusion sequences are typically performed at baseline and after therapy in order to evaluate radiological response. In this study, we plan to add Ga68-NEB (Gallium-68 NOTA-Evans Blue) PET imaging and DCE-MRI imaging to examine BBB permeability and assess in-vivo drug effects on tumor vasculature over time. These studies are planned at 3 timepoints: 1) During the pre-screening phase, following confirmation of histological diagnosis and prior to chemoradiotherapy and 2) during the main study, prior to and 8 weeks (+/- 1 week) after QPOP-guided systemic therapy. Radiological biomarkers will be correlated with radiological response based on follow up with routine MRI and clinical outcomes as well as therapeutic sensitivities.

Radiomics

MRIs obtained at initial diagnosis and subsequent scans will be subjected to radiomic feature extraction, and correlated with spatially annotated histopathological biopsies opportunistically obtained as part of standard-of-care surgical resection both at initial diagnosis (chemotherapy- and radiotherapy-naïve) and at recurrence. Annotation of biopsy site would be done on T1-stereotaxy and pathology images would be correlated with spatial radiomic features and clustering. In addition, part of the biopsy will be used to generate high grade astrocytic glioma organoids, and tested for chemosensitivity towards both temozolomide and the optimal drug combination the participant will

be treated with. These spatial properties of the high grade astrocytic glioma biopsies will be used for model building for prediction of post-chemotherapy recurrence.

b) Histopathology

Paraffin-embedded blocks of high grade astrocytic glioma tumour will be stained to assess DNA damage response (ɣH2AX, RAD51), growth factor receptors (EGFR, PDGFR) and hypoxia markers (HIF1A) in relation to blood vessels and axons. Molecular analyses of TP53, ATRX, IDH1, IDH2, BRAF, and EGFRvIII mutations, MGMT methylation status, and 1p19q co-deletion would be performed as part of routine care.

c) Plasma biomarkers

Approximately Fifteen to Eighteen millilitres of blood will be collected together with routine blood tests for plasma biomarkers including circulating tumor DNA, proteomics and DNA methylation at these time points. These will be stored in the NUH tissue repository for future analysis.

- Prescreening - At first MRI scan before chemoradiotherapy
- Main study – At baseline and 8-week post QPOP-guided chemotherapy

9 SAFETY MEASUREMENTS

9.1 Definitions

__**Adverse Event**__

An adverse event is defined as any untoward medical occurrence in a subject administered a pharmaceutical product and which does not necessarily have a causal relationship with the treatment. An adverse event can therefore be any unfavorable and unintended sign (including an abnormal laboratory finding), symptom, or disease temporally associated with the use of a medicinal (investigational) product, whether or not the event is considered causally related to the use of the product. Such an event can result from use of the drug as stipulated in the protocol or labelling, as well as from accidental or intentional overdose, drug abuse, or drug withdrawal. Any worsening of a pre-existing condition or illness is considered an adverse event (with the exception of cancer). Clinical signs and symptoms of disease progression are considered adverse events. Laboratory abnormalities and changes in vital signs are considered to be adverse events only if they result in discontinuation from the study, necessitate therapeutic medical intervention, meet protocol specific criteria and/or if the investigator considers them to be adverse events.

CTCAE term (adverse event description) and grade: The descriptions and grading scales found in the revised NCI Common Terminology Criteria for Adverse Events (CTCAE) version 5.0 will be utilized for adverse event reporting. A copy of the CTCAE version 5.0 can be downloaded from the CTEP web site (http://ctep.cancer.gov/reporting/ctc.html).

• **"Expectedness"**: Adverse events can be "Expected" or unexpected.
• **Attribution** of the adverse event:
- Definite – The adverse event *is clearly related* to the study treatment.
- Probable – The adverse event *is likely related* to the study treatment.
- Possible – The adverse event *may be related* to the study treatment.
- Unlikely – The adverse event *is doubtfully related* to the study treatment.
- Unrelated – The adverse event *is clearly NOT related* to the study treatment.

Serious Adverse Event

A serious adverse event is any untoward medical occurrence at any dose that meets any of the following criteria, whether related to study drug or not:

Death of Subject- An event that results in the death of a subject.

Life-Threatening- An event that, in the opinion of the investigator, would have resulted in immediate fatality if medical intervention had not been taken. This does not include an event that would have been fatal if it had occurred in a more severe form.

Hospitalization- An event that results in an admission to the hospital for any length of time. This does not include an emergency room visit or admission to an out-patient facility.

Prolongation of Hospitalization- An event that occurs while the study subject is hospitalized and prolongs the subject's hospital stay.

Congenital Anomaly- An anomaly detected at or after birth, or any anomaly that results in fetal loss.

Persistent or Significant Disability/Incapacity- An event that results in a condition that substantially interferes with the activities of daily living of a study subject. Disability is not intended to include experiences of relatively minor medical significance such as headache, nausea, vomiting, diarrhoea, influenza, and accidental trauma.

Important Medical Event Requiring Medical or Surgical Intervention to Prevent Serious Outcome An important medical event that may not be immediately life-threatening or result in death or hospitalization, but based on medical judgment may jeopardize the subject and may require medical or surgical intervention to prevent any of the outcomes listed above (*i.e.*, death of subject, life-threatening, hospitalization, prolongation of hospitalization, congenital anomaly, or persistent or significant disability/incapacity). Examples of such events include allergic bronchospasm requiring intensive treatment in an emergency room or at home, blood dyscrasias or convulsions that do not result in inpatient hospitalization, or the development of drug dependency or drug abuse.

Spontaneous Abortion Miscarriage experienced by study subject.

Elective Abortion Elective abortion performed on study subject.

9.2 Collecting, Recording and Reporting of "Unanticipated Problems Involving Risk to Subjects or Others" – UPIRTSO events to the NHG Domain Specific Review Boards (DSRB)

UPIRTSO events refers to problems, in general, to include any incident, experience, or outcome (including adverse events) that meets ALL of the following criteria:

1. **Unexpected**
   In terms of nature, severity or frequency of the problem as described in the study documentation (eg: Protocol, Consent documents etc).

2. **Related or possibly related to participation in the research**
   Possibly related means there is a reasonable possibility that the problem may have been caused by the procedures involved in the research; and

3. **Risk of harm**
   Suggests that the research places participants or others at a greater risk of harm (including physical, psychological, economic, or social harm) than was previously known or recognized.

Reporting Timelines for UPIRTSO Events to the NHG DSRB:

1. **Urgent Reporting**: All problems involving local deaths, whether related or not, should be reported immediately – within 24 hours after first knowledge by the investigator.

2. **Expedited Reporting:** All other problems must be reported as soon as possible but not later than 7 calendar days after first knowledge by the investigator.

9.3 Safety Monitoring Plan

The study team will meet to review the QPOP analysis results, agree on the validity of the data and determine the recommended treatment at the time of relapse for each patient enrolled onto the study. The investigator is responsible for appropriate medical care of subjects during the study. Investigator will review all patients before each treatment cycle for monitoring of toxicity. If a subject reports an adverse event, the investigator should follow the subject until the event is either resolved or assessed as stable. Safety data will be monitored every week by reviewing the toxicity data by the principal investigator, at least one other investigator and the research nurse. All SAE will be reviewed. Collection period of SAE is from first QPOP-guided study dosing until 28 days after discontinuation.

As an additional safety measure, the study team will also conduct a combined review for each patient at the time of their first post-treatment radiologic assessment, to ensure the appropriateness of therapy based on the efficacy and toxicity of the treatment selected. Interim analysis of the PFS6 will be performed after 5 patients have received QPOP-guided chemotherapy. If PFS6 <15% (i.e., none of the patients have achieved 6 months of progression-free survival), this indicates that the trial will likely be futile.[34] The study team will then meet to review potential reasons for this and decide if the trial should be discontinued.

10 DATA ANALYSIS

10.1 Data Quality Assurance

Although a research nurse or study coordinator may interview subjects and complete the case report forms, the investigator will verify that all data entries are accurate and correct, including verification that the subject fulfils the criteria for entry into the study before study medication is dispensed. Physical examinations have to be performed by a registered medical practitioner. All relevant forms including End of Treatment Forms must be completed for each patient upon completion or withdrawal from the study.

10.2 Data Entry and Storage

All data obtained in the study described in this protocol will be recorded on electronic case report forms (eCRFs) named REDCAP. The eCRFs will be completed chronologically and updated regularly in order to reflect the most recent data on the patient included in the study.

The investigators of the trial will be responsible for the integrity and accuracy of the data collected. All electronic data will be kept in system and only authorised study team personnel with Redcap access can access the data. Prior to the start of the study, the Principal Investigator will delegate personnel who are authorized to make or change entries on the eCRFs.

Each eCRF pages must be completely filled by referring to study source documents. All the eCRF pages which include, but not limited to the registration form, the treatment forms and the follow-up status form must be reviewed by authorized investigator throughout the study conduct.

Although a study co-ordinator may interview subjects, the investigator must verify that all data entries are accurate and correct, including verification that the subject fulfils the criteria for entry into the study. Physical examinations have to be performed by a registered medical practitioner.

The End of Treatment Form must be completed for each patient upon completion or withdrawal from the study.

11 SAMPLE SIZE AND STATISTICAL METHODS

This is an interventional, non-randomized, single site study. The primary purpose of this study is not hypothesis testing, but to assess the feasibility of QPOP-guided therapy for recurrent high grade astrocytic glioma to be used in a larger scale study. Therefore, this study does not have a formal sample size, but rather, a set benchmarks to determine feasibility. We plan to enrol a total of 10 patients who receive QPOP-guided chemotherapy at the time of first high grade astrocytic glioma recurrence. We expect this to be sufficient for determining the feasibility of a phase II trial in which there will be formal sample size calculations for the achievement of PFS6 $\geq$ 30%.[35] In addition, a phase II study will only be considered feasible if there is successful organoid generation and QPOP analysis in > 50% of samples collected. In this study, subjects will be replaced accordingly if their QPOP analysis is unsuccessful or its results do not lead to therapeutic decision making.

12 ETHICAL CONSIDERATIONS

12.1 Informed Consent

Obtaining and documenting informed consent will be performed by the Investigator or his/her representative in compliance with the ICH GCP guidelines and the ethical principles that have their origin in the Declaration of Helsinki. Prior to the initiation of any screening or study-specific procedures, the investigator or his/her representative will explain the nature of the study to the subject and answer all questions regarding this study. The patient will be told of his or her right to withdraw from the study at any time without any penalty with regards to the continuation of care at this institution and by the same physicians chosen by him. Each informed consent will be reviewed, signed and dated by the subject and the person who administered the informed consent. A copy of each informed consent will be given to the subject and each original will be kept in the Investigator Site File.

12.2 IRB Review

Each participating institution must provide for the review and approval of this protocol and the associated informed consent documents by the IRB / NHG DSRB.

Good Clinical Practice (GCP) requires that the clinical protocol, the Investigator's Brochure, the informed consent and all other forms of subject information related to the study (*e.g.*, advertisements used to recruit subjects) and any other necessary documents be reviewed by an IRB / NHG DSRB. IRB / NHG DSRB approval of the protocol, informed consent and subject information and/or advertising, as relevant, will be obtained prior to study commencement. Any amendments to the protocol will require IRB / NHG DSRB approval prior to implementation of any changes made to the study design. During the conduct of the study, the investigator will promptly provide written reports (*e.g.,* ICH Expedited Reports or any additional reports required by local regulations) to IRB / NHG DSRB of any changes that affect the conduct of the study and/or increase the risk to subjects.

12.3 Confidentiality of Data and Patient Records

Information collected for this study will be kept confidential. This confidentiality is extended to cover testing of biological samples and genetic tests in addition to the clinical information relating to the participating subjects. Patient records, to the extent of the applicable laws and regulations, will

not be made publicly available. Participants will be identified by codes and the information that links the codes to the subjects' identities will be stored separately. Data collected and entered into the Case Report Forms are the property of National University Hospital. In the event of any publication regarding this study, patient identity will remain confidential.

By signing the Informed Consent Form, subject (or subject's legally acceptable representative, if relevant) is authorizing (i) collection, access to, use and storage of subject's "Personal Data, and (ii) disclosure to authorized service providers and relevant third parties. "Personal Data" means data about the subject which makes him/her identifiable (i) from such data or (ii) from that data and other information which an organization has or likely to have access. This includes medical conditions, medications, investigations and treatment history. Research arising in the future, based on this Personal Data, will be subject to review by the relevant institutional review board. By participating in this research study, subject confirms that he/she has read, understood and consent to the Personal Data Protection Notification available at https://www.nuh.com.sg/Pages/Personal-Data-Protection-Act.aspx . The National University Hospital will take appropriate steps to ensure compliance with the data protection requirements in the Personal Data Protection Act while subject's 'Personal Data" to be transferred remains in its possession or under its control.

13 RETENTION OF TRIAL DOCUMENTS

Records for all participants, including Case Report Forms, all source documentation (containing evidence to study eligibility, history and physical findings, laboratory data, results of consultations, etc.) as well as IRB records and other regulatory documentation will be retained by the Principal Investigator in a secure storage facility. The records will be accessible for audit/study review by authorized authorities/Ethics Committee. All records will be kept for a period of 6 years following the date of study closure according to local requirements.

14 REFERENCES

1 Stupp, R. *et al.* Radiotherapy plus concomitant and adjuvant temozolomide for glioblastoma. *N Engl J Med* **352**, 987-996, doi:10.1056/NEJMoa043330 (2005).

2 Brandes, A. A. *et al.* First-Line Chemotherapy With Cisplatin Plus Fractionated Temozolomide in Recurrent Glioblastoma Multiforme: A Phase II Study of the Gruppo Italiano Cooperativo di Neuro-Oncologia. *Journal of Clinical Oncology* **22**, 1598-1604, doi:10.1200/jco.2004.11.019 (2004).

3 Chamberlain, M. C. & Tsao-Wei, D. D. Salvage chemotherapy with cyclophosphamide for recurrent, temozolomide-refractory glioblastoma multiforme. *Cancer* **100**, 1213-1220, doi:10.1002/cncr.20072 (2004).

4 Chatzellis, E. *et al.* Activity and Safety of Standard and Prolonged Capecitabine/Temozolomide Administration in Patients with Advanced Neuroendocrine Neoplasms. *Neuroendocrinology* **109**, 333-345, doi:10.1159/000500135 (2019).

5 Field, K. M. *et al.* Randomized phase 2 study of carboplatin and bevacizumab in recurrent glioblastoma. *Neuro Oncol* **17**, 1504-1513, doi:10.1093/neuonc/nov104 (2015).

6 Forsyth, P. *et al.* Phase II trial of docetaxel in patients with recurrent malignant glioma: a study of the National Cancer Institute of Canada Clinical Trials Group. *Invest New Drugs* **14**, 203-206, doi:10.1007/bf00210791 (1996).

7 Francesconi, A. B. *et al.* Carboplatin and etoposide combined with bevacizumab for the treatment of recurrent glioblastoma multiforme. *J Clin Neurosci* **17**, 970-974, doi:10.1016/j.jocn.2009.12.009 (2010).

8 Friedman, H. S. *et al.* Irinotecan therapy in adults with recurrent or progressive malignant glioma. *J Clin Oncol* **17**, 1516-1525, doi:10.1200/JCO.1999.17.5.1516 (1999).

9 Guidi, M. *et al.* P01.044 Impact of the addition of vinorelbine to temozolomide in the first-line treatment of pediatric high grade gliomas. *Neuro-Oncology* **20**, iii238-iii239, doi:10.1093/neuonc/noy139.086 (2018).

10 Lu, G. *et al.* Triple-drug Therapy With Bevacizumab, Irinotecan, and Temozolomide Plus Tumor Treating Fields for Recurrent Glioblastoma: A Retrospective Study. *Front Neurol* **10**, 42, doi:10.3389/fneur.2019.00042 (2019).

11 Wick, W. *et al.* Lomustine and Bevacizumab in Progressive Glioblastoma. *N Engl J Med* **377**, 1954-1963, doi:10.1056/NEJMoa1707358 (2017).

12 Wick, W. *et al.* NOA-04 randomized phase III trial of sequential radiochemotherapy of anaplastic glioma with procarbazine, lomustine, and vincristine or temozolomide. *J Clin Oncol* **27**, 5874-5880, doi:10.1200/JCO.2009.23.6497 (2009).

13 Wick, W. *et al.* Phase III study of enzastaurin compared with lomustine in the treatment of recurrent intracranial glioblastoma. *J Clin Oncol* **28**, 1168-1174, doi:10.1200/JCO.2009.23.2595 (2010).

14 Wen, P. Y. *et al.* Updated response assessment criteria for high-grade gliomas: response assessment in neuro-oncology working group. *J Clin Oncol* **28**, 1963-1972, doi:10.1200/JCO.2009.26.3541 (2010).

15 Wesseling, P. & Capper, D. WHO 2016 Classification of gliomas. *Neuropathol Appl Neurobiol* **44**, 139-150, doi:10.1111/nan.12432 (2018).

16 Wenger, A. *et al.* Intratumor DNA methylation heterogeneity in glioblastoma: implications for DNA methylation-based classification. *Neuro Oncol* **21**, 616-627, doi:10.1093/neuonc/noz011 (2019).

17 Qazi, M. A. *et al.* Intratumoral heterogeneity: pathways to treatment resistance and relapse in human glioblastoma. *Ann Oncol* **28**, 1448-1456, doi:10.1093/annonc/mdx169 (2017).

18 Ellis, H. P. *et al.* Current Challenges in Glioblastoma: Intratumour Heterogeneity, Residual Disease, and Models to Predict Disease Recurrence. *Front Oncol* **5**, 251, doi:10.3389/fonc.2015.00251 (2015).

19 Gillet, J. P., Varma, S. & Gottesman, M. M. The clinical relevance of cancer cell lines. *J Natl Cancer Inst* **105**, 452-458, doi:10.1093/jnci/djt007 (2013).

20 Ledford, H. US cancer institute to overhaul tumour cell lines. *Nature* **530**, 391, doi:10.1038/nature.2016.19364 (2016).

21 Virtanen, C. *et al.* Integrated classification of lung tumors and cell lines by expression profiling. *Proc Natl Acad Sci U S A* **99**, 12357-12362, doi:10.1073/pnas.192240599 (2002).

22 Hubert, C. G. *et al.* A Three-Dimensional Organoid Culture System Derived from Human Glioblastomas Recapitulates the Hypoxic Gradients and Cancer Stem Cell Heterogeneity of Tumors Found In Vivo. *Cancer Res* **76**, 2465-2477, doi:10.1158/0008-5472.CAN-15-2402 (2016).

23 Jacob, F. *et al.* A Patient-Derived Glioblastoma Organoid Model and Biobank Recapitulates Inter- and Intra-tumoral Heterogeneity. *Cell* **180**, 188-204 e122, doi:10.1016/j.cell.2019.11.036 (2020).

24 Vlachogiannis, G. *et al.* Patient-derived organoids model treatment response of metastatic gastrointestinal cancers. *Science* **359**, 920-926, doi:10.1126/science.aao2774 (2018).

25 Shaughnessy, J. D., Jr. *et al.* Pharmacogenomics of bortezomib test-dosing identifies hyperexpression of proteasome genes, especially PSMD4, as novel high-risk feature in myeloma treated with Total Therapy 3. *Blood* **118**, 3512-3524, doi:10.1182/blood-2010-12-328252 (2011).

26 Rashid, M. *et al.* Optimizing drug combinations against multiple myeloma using a quadratic phenotypic optimization platform (QPOP). *Sci Transl Med* **10**, doi:10.1126/scitranslmed.aan0941 (2018).

27 Tan, D. *et al.* Panobinostat in combination with bortezomib in patients with relapsed or refractory peripheral T-cell lymphoma: an open-label, multicentre phase 2 trial. *Lancet Haematol* **2**, e326-333, doi:10.1016/S2352-3026(15)00097-6 (2015).

28 de Mel, S. *et al.* Application of an ex-vivo drug sensitivity platform towards achieving complete remission in a refractory T-cell lymphoma. *Blood Cancer J* **10**, 9, doi:10.1038/s41408-020-0276-7 (2020).

29 Ronaldson, P. T. & Davis, T. P. Targeting blood-brain barrier changes during inflammatory pain: an opportunity for optimizing CNS drug delivery. *Ther Deliv* **2**, 1015-1041, doi:10.4155/tde.11.67 (2011).

30 Zhang, J. *et al.* Clinical Translation of an Albumin-Binding PET Radiotracer 68Ga-NEB. *J Nucl Med* **56**, 1609-1614, doi:10.2967/jnumed.115.159640 (2015).

31 Long, X. *et al.* Microsurgery guided by sequential preoperative lymphography using (68)Ga-NEB PET and MRI in patients with lower-limb lymphedema. *Eur J Nucl Med Mol Imaging* **44**, 1501-1510, doi:10.1007/s00259-017-3676-6 (2017).

32 Zhang, W. *et al.* Potential Applications of Using 68Ga-Evans Blue PET/CT in the Evaluation of Lymphatic Disorder: Preliminary Observations. *Clin Nucl Med* **41**, 302-308, doi:10.1097/RLU.0000000000001171 (2016).

33 The 2007 Recommendations of the International Commission on Radiological Protection. ICRP publication 103. *Ann ICRP* **37**, 1-332, doi:10.1016/j.icrp.2007.10.003 (2007).

34 Polley, M. Y. *et al.* Six-month progression-free survival as an alternative primary efficacy endpoint to overall survival in newly diagnosed glioblastoma patients receiving temozolomide. *Neuro Oncol* **12**, 274-282, doi:10.1093/neuonc/nop034 (2010).

35 Mandel, J.J. *et al.* Inability of positive phase II clinical trials of investigational treatments to subsequently predict positive phase III clinical trials in glioblastoma. *Neuro Oncol* **20**, 113-122, doi: 10.1093/neuonc/nox144.

15 Appendix 1 Study Schedule

Assessment/Procedures	Pre-screening			Main study		
	Before surgery	During surgery	Before chemoradiotherapy[e]	Pre-treatment (within 2 weeks)	At the start of each cycle thereafter	End of treatment [a]
Obtaining of informed consent	X			X		
Medical history				X	X	X
Physical examination				X		X
Concomitant medication notation				X		X
ECOG Performance status				X		X
Toxicity assessments (CTCAE v 5.0)						X
Haematology (Full blood count)				X	X (±3 days)	
Blood chemistry [b]				X		
Serum pregnancy test[d]				X		
Radiologic tumour measurement [c]			X	X	X (8 weeks (+/-1 week) after QPOP-guided systemic therapy)	
Blood Sampling for research purposes (performed together with routine blood tests)			X	X	X (8 weeks (+/-1 week) after QPOP-guided systemic therapy)	
Tumor sample collection[f]		X		X [g]		

Note:

a. Final study visit procedures can be done within 30 days of study discontinuation/completion of study treatment
b. Includes bilirubin, ALT, AST, ALP, creatinine, calcium, sodium, potassium
c. Standard MRI brain with contrast (Ga68-NEB PET/MRI and DCE-MRI) prior to chemoradiotherapy (Radiological timepoint 1), prior to and 8 weeks (+/-1 week) after QPOP-guided

systemic therapy (Radiological timpepoints 2 and 3) for evaluation of tumor response. Subsequent to this, radiological assessment will revert to standard clinical protocols with routine standard MRI imaging.

d. Applicable only for female participants or women of childbearing potential.
e. Prior to chemoradiotherapy
f. If surgery is deemed to be too risky for patient at pre-screening phase, patient biopsy sample will be used instead if there is sufficient tumor tissue. In addition, tumor may be harvested for any subsequent procedure that patient might require for brain tumor treatment.
g. If there is a second tissue collection at relapse.
